# Supplementary material for: Treatment of tubular damage in high-fat-diet-fed obese mice using sodium-glucose co-transporter inhibitors
Source: PLoS One. 2023 Feb 13;18(2):e0281770. doi: 10.1371/journal.pone.0281770 (PMC9925073; doi:10.1371/journal.pone.0281770)
Supplement: S1 File — (DOCX) [file pone.0281770.s001.docx]

**Supporting Information**

**
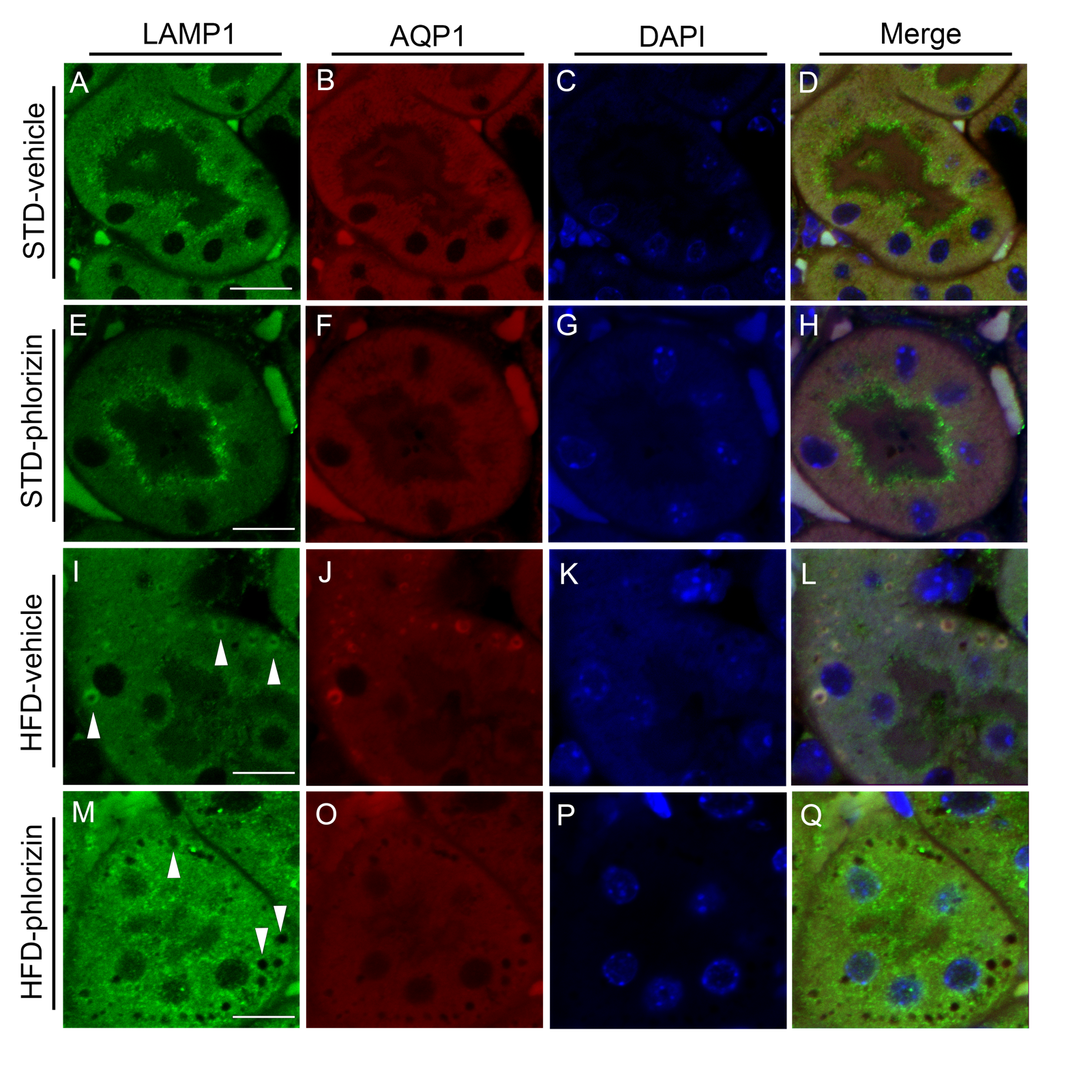
**

**S1 Fig. Immunostaining of lysosomal marker Lamp1 in kidney proximal tubule epithelial cells in the HFD-PLZ assay**. In STD-vehicle and STD-phlorizin groups, LAMP1 was stained as dots at the apical side (A, D, E, and H). In HFD-vehicle, LAMP1 was stained in a toroidal shape as lamellar bodies (I arrowheads and L). In HFD-PLZ, vacuoles were mainly on the basal side (M arrowheads), and LAMP1 was stained as dots throughout (M and Q). AQP1 was stained as a marker for the proximal tubule (B, F, J, and O). DAPI was stained in the nucleus (C, G, K, and P). Merged images (D, H, L, and Q). Bars; (A, E, I, and M) 10 μm.

**
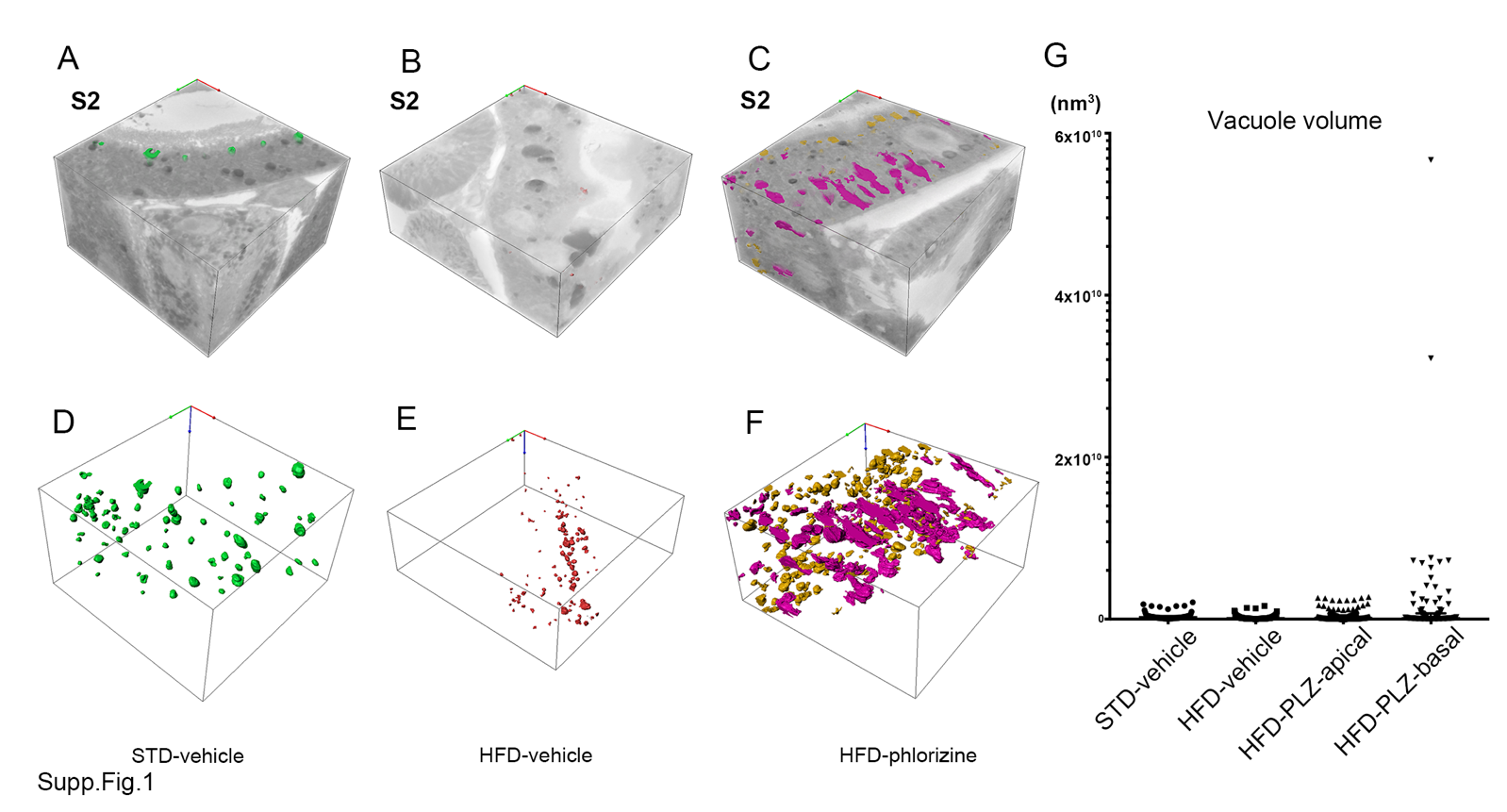
**

**S2 Fig. Vacuolar changes occurring in segment 2 of the proximal tubules in obese mice fed with a high**-**fat diet (HFD) after phlorizin (PLZ) administration**. (A–C) Segmentation of vacuoles merged on the 3D-reconstructed SBF-SEM image of the HFD-phlorizin assay model. (D–F) 3D reconstructed image of a segmented vacuole. (A, D) Standard diet (STD)-vehicle (vacuole; green), (B, E) HFD-vehicle (vacuole; orange), (C, D) HFD-phlorizin (apical vacuole; yellow, basal vacuole; red). (G) Graph of vacuolar volume in the segmentation image of SBF-SEM (D–F). PLZ, phlorizin; Veh, vehicle; HFD, high-fat diet; STD, standard diet.

**
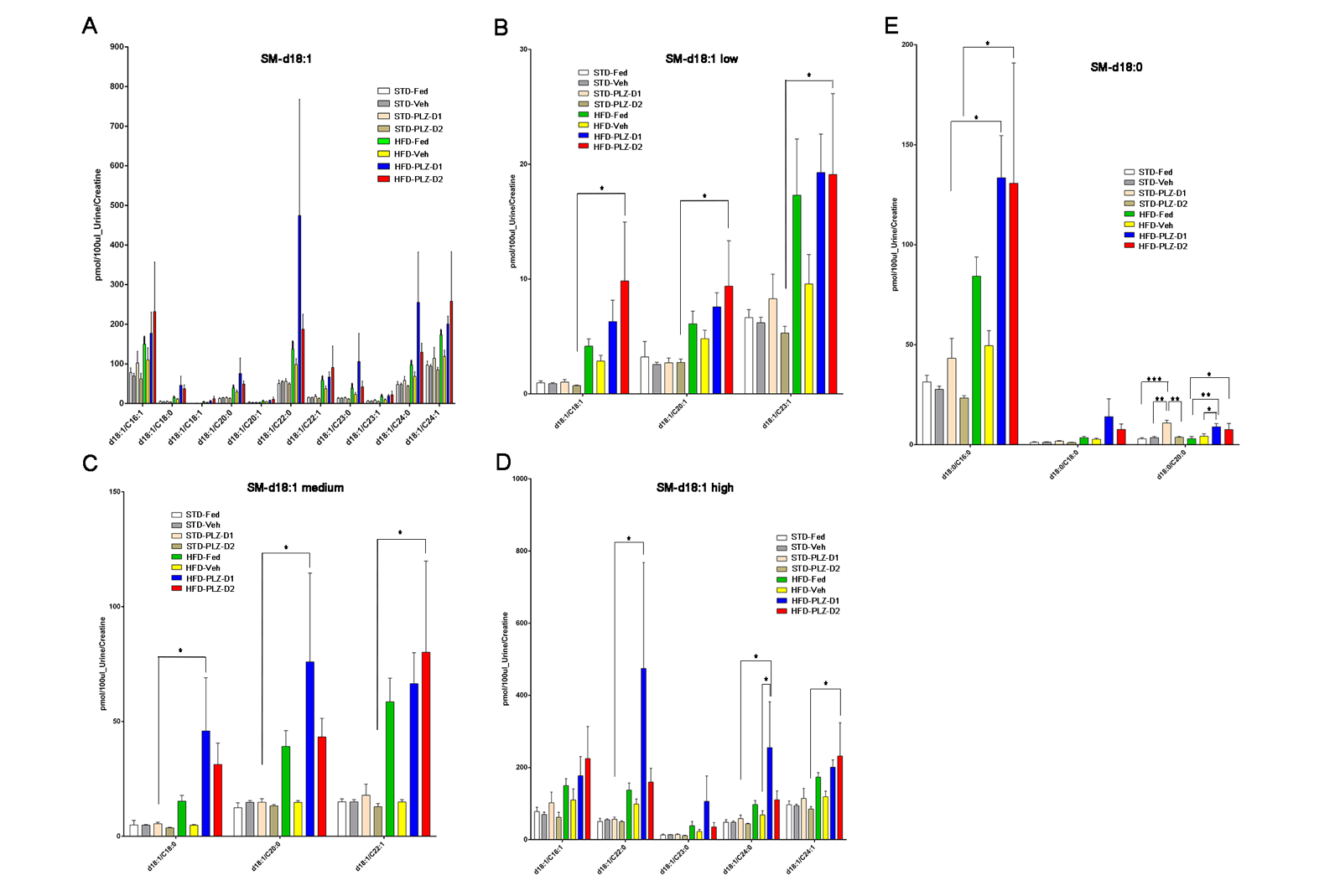
**

**S3 Fig. Liquid chromatography-tandem mass spectrometry (LC-MS/MS) was used to quantify sphingomyelin levels in sphingomyelin (SM) (d18:0) and SM (d18:1) in the urinary extracts of sphingomyelin from the kidneys of the standard diet (STD)- and high**-**fat diet (HFD)-fed mice in the phlorizin (PLZ) assay**. LC-MS/MS was used to quantify sphingomyelin levels from (A) SM(d18:1), (B) low-intensity SM (d18:1), (C) medium intensity SM (d18:1), (D) high intensity SM (d18:1), and (E) SM (d18:0). (A–D) When sphingomyelin was analyzed separately for d18:0 and d18:1 with a focus on d18:1, the profile indicated that the SM (18:1) species C18:1, C20:1, and C23:1 had low intensity; C18:0, C22:0, and C22:1 had medium intensity; and C16:1, C22:0, C23:0, C24:0, and C24.1 had high intensity. Urine was obtained from C57/BL6J mice fed with a STD (STD-Fed) or HFD (HFD-Fed) prior to vehicle administration, vehicle-treated mice (Veh) prior to phlorizin or vehicle administration, phlorizin- or vehicle-treated mice on day one (PLZ-D1 or Veh-D1), and phlorizin- or vehicle-treated mice on day two (PLZ-D2 or Veh-D2). PLZ, phlorizin; Veh, vehicle; HFD, high-fat diet; STD, standard diet. Results are presented as the mean ± standard deviation. **P* < 0.05, ***P* < 0.01, ****P* < 0.001. Statistical significance was calculated by two-way analysis of variance followed by the post-hoc Tukey–Kramer test and Sidak’s multiple comparison test.


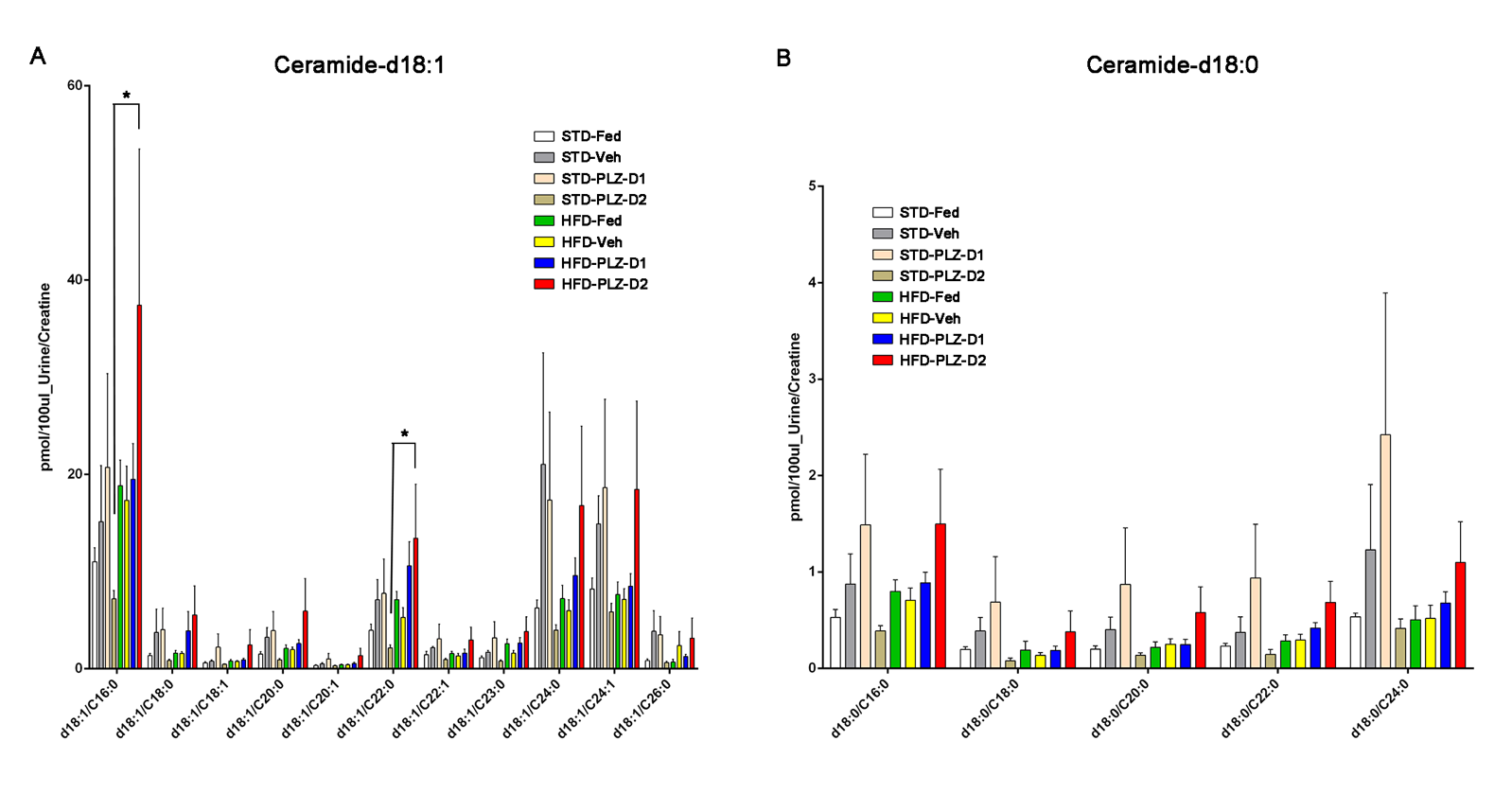


**S4 Fig. Liquid chromatography**-**tandem mass spectrometry was used to quantify ceramide (Cer) levels from Cer (d18:0) and Cer (d18:1) in the urinary extracts of sphingomyelin from the kidneys of the standard diet (STD)- and HFD-fed mice in the phlorizin (PLZ) assay**. When ceramides were analyzed separately for d18:1 (A) and d18:0 (B), focusing on ceramide (d18:1), the profiles of C16:0 and C22:0 species were significantly higher in HFD-PLZ-day 2 than in STD-PLZ-day 2. Urine was obtained fromC57/BL6J mice fed with a STD (STD-Fed) or HFD (HFD-Fed) prior to vehicle administration, vehicle-treated mice (Veh) prior to phlorizin or vehicle administration, phlorizin- or vehicle-treated mice on day one (PLZ-D1 or Veh-D1), and phlorizin- or vehicle-treated mice on day two (PLZ-D2 or Veh-D2). PLZ, phlorizin; Veh, vehicle; HFD, high-fat diet; STD, standard diet. Results are presented as the mean ± standard deviation. **P* < 0.05. Statistical significance was calculated by two-way analysis of variance followed by the post-hoc Tukey–Kramer test and Sidak’s multiple comparison test.


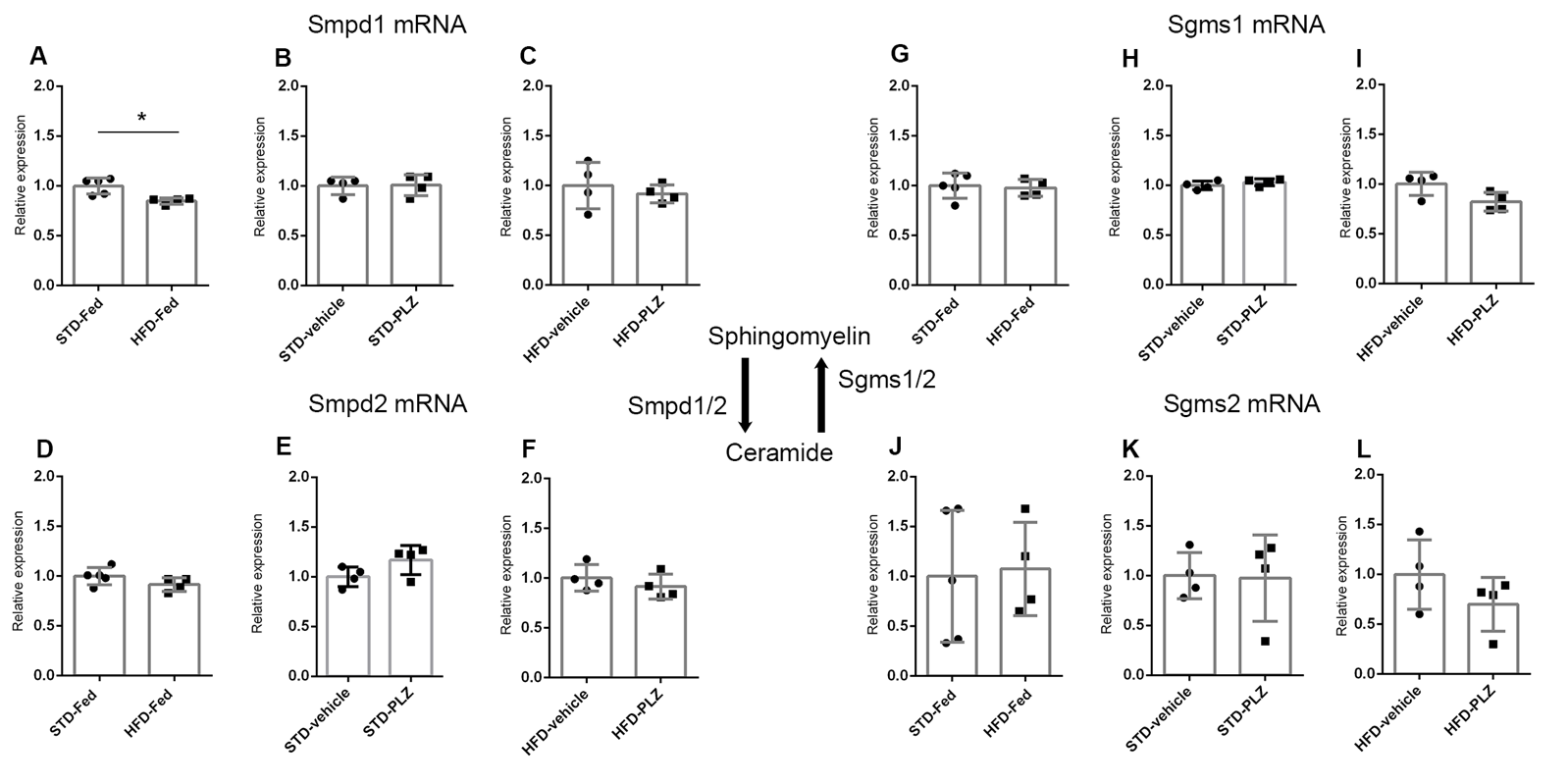


**S5 Fig. Changes in the expression levels of genes associated with sphingomyelinase and sphingomyelin synthesis in phlorizin assays.** (A–C) Relative expression of *Smpd1* mRNA. (D–F) Relative expression of *Smpd2* mRNA. (G–I) Relative expression of *Sgms1* mRNA. (J–L) Relative expression of *Sgms2* mRNA.


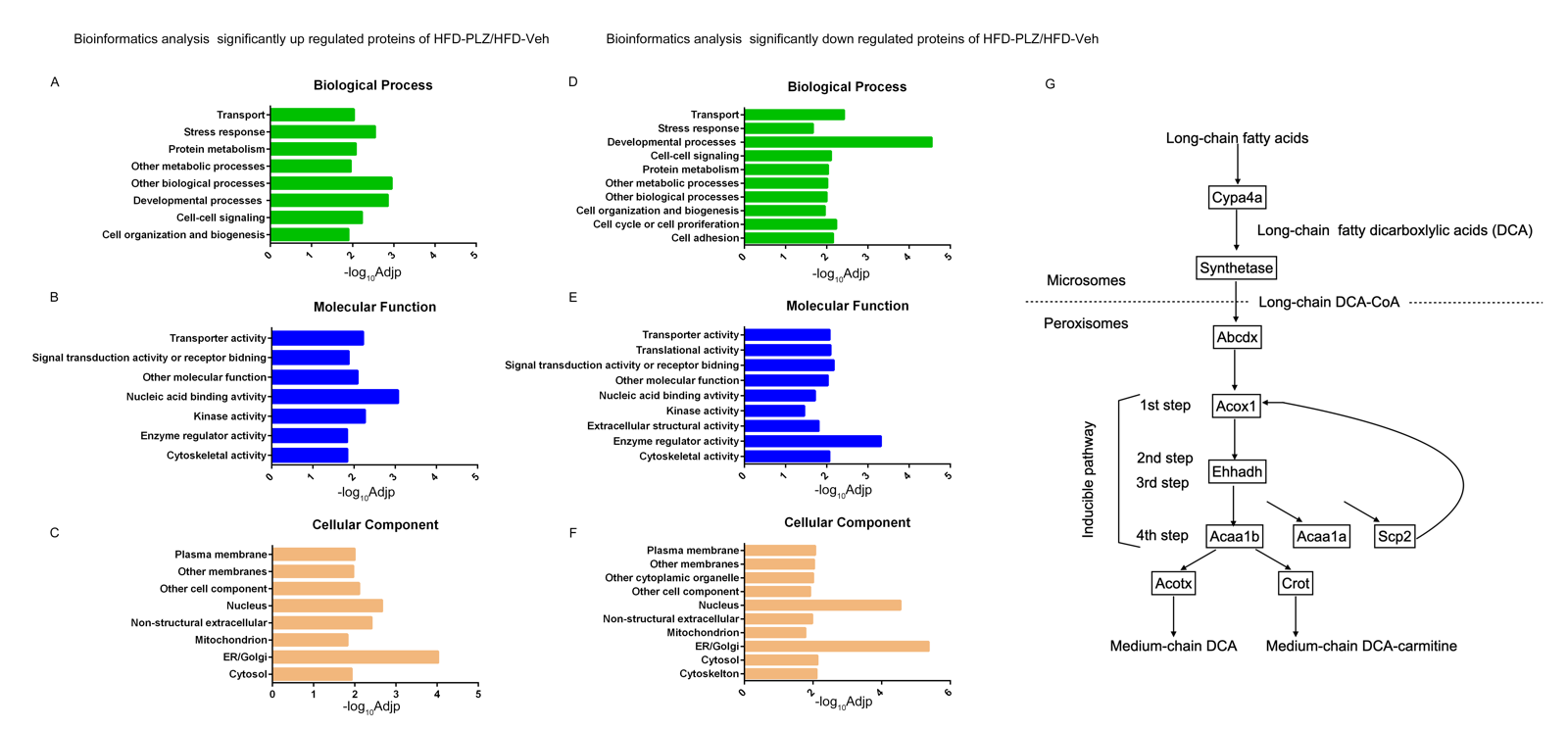


**S6 Fig. Gene Ontology annotation of differentially expressed proteins between high-fat diet (HFD)-phlorizin and HFD-vehicle mice and scheme of the four enzymes of the peroxisomal beta-oxidation pathway**. (A–C) Bioinformatics analysis of significantly upregulated proteins. (A) Analysis of biological processes. (B) Analysis of molecular function. (C) Analysis of cellular components. (D–E) Bioinformatics analysis of significantly downregulated proteins. (D) Analysis of biological processes. (E) Analysis of molecular function. (F) Analysis of cellular components. (G) Schematic representation of long-chain fatty acid ω-oxidation and subsequent peroxisomal long-chain dicarboxylic acid (DCA) β-oxidation.

**S1** **Table.** **List of primers used in real-time PCR.**

| **Gene** | **Description** | **Sequence** |
| --- | --- | --- |
| *Smpd1* | Forward | 5'-TGGGACTCCTTTGGATGGG-3' |
|  | Reverse | 5'-CGGCGCTATGGCACTGAAT-3' |
| *Smpd2* | Forward | 5'-CTCGCCGCCCTTGCT-3' |
|  | Reverse | 5'-CAGCCGTAGAGAAAAGTTGAGCTT-3' |
| *Sgms1* | Forward | 5'-ACCACCGTGTGCTTCTGTATCCTA-3' |
|  | Reverse | 5'-AAATGGCTTACAGCCCTGTCTTTG-3' |
| *Sgms2* | Forward | 5'-GATTACTTCGACCGGGTCAA-3' |
|  | Reverse | 5'-GCACAGGTAACGTAGTGACA-3' |
| *Gapdh* | Forward | 5'-TGTGTCCGTCGTGGATCTGA-3' |
|  | Reverse | 5'-GGTCCTCAGTGTAGCCCAAG-3' |

**S2 Table. Fatty acid metabolism and sphingolipid metabolism.**

1. Fatty acid biosynthesis

| Gene name | Abundance Ratio | | Abundance Ratio  Adj. P-Value | |
| --- | --- | --- | --- | --- |
|  | HFD-PLZ/ HFD-Veh | HFD-Veh/  STD-Veh | HFD-PLZ/  HFD-Veh | HFD-Veh/ STD-Veh |
| Oxsm | 1.007 | 0.944 | N.S. | N.S. |
| Acsl1 | 1.086 | 0.904 | N.S. | N.S. |
| Acsl5 | 1.048 | 0.956 | N.S. | N.S. |
| Mecr | 1.087 | 0.882 | N.S. | N.S. |
| Cbr4 | 0.937 | 0.897 | N.S. | N.S. |
| Mcat | 1.009 | 0.913 | N.S. | N.S. |
| Fasn | 1.091 | 0.602 | N.S. | 0.00050655 |
| Acaca | 0.991 | 0.675 | N.S. | N.S. |
| Hsd17b8 | 1.011 | 0.879 | N.S. | N.S. |
| Acsf3 | 1.032 | 0.864 | N.S. | N.S. |

1. Fatty acid elongation

| Gene name | Abundance Ratio | | Abundance Ratio  Adj. P-Value | |
| --- | --- | --- | --- | --- |
|  | HFD-PLZ/ HFD-Veh | HFD-Veh/  STD-Veh | HFD-PLZ/ HFD-Veh | HFD-Veh/ STD-Veh |
| Hacd3 | 1.182 | 0.866 | N.S. | N.S. |
| Tecr | 1.044 | 0.951 | N.S. | N.S. |
| Elovl1 | 1.134 | 1.297 | N.S. | N.S. |
| Acot1 | 1.191 | 0.964 | N.S. | N.S. |
| Acot2 | 1.295 | 1.053 | 0.02210003 | N.S. |
| Acot3 | 1.757 | 0.981 | N.S. | N.S. |
| Acot4 | 0.9 | 0.953 | N.S. | N.S. |
| Echs1 | 1.046 | 0.87 | N.S. | N.S. |
| Hadha | 1.249 | 0.858 | N.S. | N.S. |
| Hadh | 1.013 | 1.181 | N.S. | N.S. |
| Acaa2 | 1.076 | 0.78 | N.S. | N.S. |
| Hadhb | 1.208 | 0.907 | N.S. | N.S. |
| Ppt1 | 1.395 | 0.956 | 0.025171 | N.S. |
| Them4 | 1.044 | 0.925 | N.S. | N.S. |
| Hsd17b12 | 1.045 | 1.048 | N.S. | N.S. |

1. Citrate cycle (TCA cycle)

| Gene name | Abundance Ratio | | Abundance Ratio  Adj. P-Value | |
| --- | --- | --- | --- | --- |
|  | HFD-PLZ/ HFD-Veh | HFD-Veh/  STD-Veh | HFD-PLZ/ HFD-Veh | HFD-Veh/ STD-Veh |
| Mdh1 | 1.14 | 1.079 | N.S. | N.S. |
| Mdh2 | 1.042 | 0.923 | N.S. | N.S. |
| Idh1 | 1.075 | 0.885 | N.S. | N.S. |
| Idh2 | 1.066 | 0.772 | N.S. | N.S. |
| Aco1 | 1.108 | 1.05 | N.S. | N.S. |
| Aco2 | 1.114 | 0.945 | N.S. | N.S. |
| Cs | 1.057 | 0.817 | N.S. | N.S. |
| Dlst | 1.004 | 0.937 | N.S. | N.S. |
| Acly | 1.053 | 1.028 | N.S. | N.S. |
| Ogdh | 1.048 | 0.936 | N.S. | N.S. |
| Idh3a | 1.117 | 0.829 | N.S. | N.S. |
| Idh3g | 1.089 | 0.875 | N.S. | N.S. |
| Sdha | 0.956 | 0.961 | N.S. | N.S. |
| Sdhb | 0.907 | 0.902 | N.S. | N.S. |
| Sdhc | 0.954 | 0.728 | N.S. | N.S. |
| Sdhd | 0.916 | 1.002 | N.S. | N.S. |
| Sucla2 | 1.086 | 0.98 | N.S. | N.S. |
| Suclg1 | 1.05 | 0.935 | N.S. | N.S. |
| Suclg2 | 1.099 | 1.402 | N.S. | 0.02022023 |
| Fh | 1.115 | 1.049 | N.S. | N.S. |

1. Sphingolipid metabolism

| Gene name | Abundance Ratio | | Abundance Ratio  Adj. P-Value | |
| --- | --- | --- | --- | --- |
|  | HFD-PLZ/ HFD-Veh | HFD-Veh/  STD-Veh | HFD-PLZ/ HFD-Veh | HFD-Veh/STD-Veh |
| Kdsr | 0.936 | 0.881 | N.S. | N.S. |
| Sphk2 | 2.038 | 0.587 | N.S. | N.S. |
| Galc | 0.889 | 1.544 | N.S. | N.S. |
| Arsa | 1.601 | 0.922 | N.S. | N.S. |
| Smpd2 | 1.01 | 1.006 | N.S. | N.S. |
| Sptlc1 | 1.031 | 0.886 | N.S. | N.S. |
| Sptlc2 | 1.043 | 1.004 | N.S. | N.S. |
| Cers6 | 0.957 | 1.043 | N.S. | N.S. |
| Sgpl1 | 1.046 | 0.949 | N.S. | N.S. |
| Asah1 | 0.933 | 0.803 | N.S. | N.S. |
| Asah2 | 1.156 | 1.338 | N.S. | N.S. |
| Degs1 | 0.948 | 1.123 | N.S. | N.S. |
| Cers2 | 1.152 | 0.887 | N.S. | N.S. |

Statistical analyses of abundance ratio performed using two-tailed Student’s *t*-test. Significant differences were determined as *P* < 0.05. N.S, Not significant in Abundance Ratio Adj. P-Value; PLZ, phlorizin; Veh, vehicle.
